# Nurses’ Work Methods Assessment Scale: Turkish Validity and Reliability Study

**DOI:** 10.3390/nursrep15060220

**Published:** 2025-06-17

**Authors:** Dilek Uysal, Nilüfer Demirsoy, Aysun Türe, Müzelfe Bıyık, Letícia de Lima Trindade, Olga Maria Pimenta Lopes Ribeiro, João Miguel Almeida Ventura-Silva

**Affiliations:** 1 Eskisehir Health Services Vocational School, Eskisehir Osmangazi University, 26040 Eskisehir, Turkey; dilekvuraluysal@gmail.com; 2 Department of History of Medicine and Medical Ethics, Faculty of Medicine, Eskisehir Osmangazi University, 26040 Eskisehir, Turkey; nilufer_p2@hotmail.com; 3 Department of Nursing Management, Faculty of Health Sciences, Eskisehir Osmangazi University, 26040 Eskisehir, Turkey; aysunnture@gmail.com; 4 Department of Nursing Management, Faculty of Health Sciences, Kutahya Health Sciences University, 43100 Eskisehir, Turkey; muzelfeuysal@gmail.com; 5 Department of Nursing, Santa Catarina State University, Florianópolis 88035-901, SC, Brazil; leticia.trindade@udesc.br; 6 Nursing School of Porto, 4200-072 Porto, Portugal; olgaribeiro@esenf.pt; 7 RISE@Health, 4200-319 Porto, Portugal; 8 Department of Nursing, Northern Health School of the Portuguese Red Cross, 3720-126 Oliveira de Azeméis, Portugal

**Keywords:** nursing care, nurses, work, nursing administration research, validation study

## Abstract

**Background/Objectives**: Organizing nurses’ work is crucial for ensuring patient care quality and efficiency. Nurses’ work methods directly influence patient safety and healthcare outcomes, making them vital for effective health services. Assessing these methods helps identify effective practices, enhance work organization, and improve both professional satisfaction and patient safety. This study aims to translate, adapt, and validate the Nurse Work Method Assessment Scale (NWMAS) for Turkish. **Methods**: Methodological study with a non-probabilistic sample of 209 hospital nurses, conducted between June and July 2024. The linguistic adaptation involved translation and back-translation with the participation of bilingual experts. Statistical analyses included exploratory and confirmatory factor analyses, item-total correlation tests, test-retest reliability, and internal consistency assessment using Cronbach’s alpha. **Results**: One item was removed due to cultural incompatibility, resulting in a 24-item Turkish version of the NWMAS. During the adaptation process, expert evaluations led to the removal of one item from the original scale, as it referenced nursing practices that are either not widely implemented or considered culturally incompatible with the structure of the Turkish healthcare system. Content Validity Index values ranged from 0.85 to 0.95. Exploratory factor analysis confirmed a five-factor structure explaining 55.65% of total variance. Confirmatory factor analysis supported this structure with acceptable fit indices (χ^2^/df = 1.89; RMSEA = 0.06; GFI = 0.86). Cronbach’s alpha for the overall scale was 0.87, with subscale alphas ranging from 0.52 to 0.82. Test-retest reliability coefficients ranged from 0.95 to 0.98, indicating high stability over time. **Conclusions**: The Turkish version of the NWMAS demonstrated adequate validity and reliability and can be used to evaluate nurses’ work methods in Turkish hospital settings. The study highlights the importance of cultural adaptation in scale development to ensure conceptual relevance in local healthcare systems.

## 1. Introduction

Nursing, as a profession, has played a predominant role in healthcare by safeguarding individual health, treating illnesses, and providing the highest level of patient care [1]. Nurses make up more than 50% of the healthcare workforce [2], playing a key role in patient care satisfaction and in influencing and reducing healthcare costs. In fact, it is a work organization approach focused on addressing patients’ human responses and preventing complications [3]. Effectively, the way nurses organize their work and provide care can impact the reduction of hospital admissions, achieve positive patient outcomes, and control the costs generated by patients within the healthcare system, promoting safe and quality healthcare [4].

Discussing the organization of nursing care necessarily involves examining nurses’ work methods. These methods are essential to meet patients’ physical, emotional, and psychological needs while also ensuring the efficiency, quality, and safety of healthcare services [5,6]. In the literature, work methods are generally defined as the way nurses organize their workloads, prioritize care processes, and utilize tools and techniques within these processes [7,8]. This concept also includes how nurses structure their workflows and the strategies they implement to ensure patient safety [9].

Thus, adopting a work method is imperative in understanding how nurses plan, organize, and deliver patient care. Assessing nurses’ work methods is therefore an important step not only for improving healthcare system effectiveness but also for enhancing patient care quality and increasing nurses’ job satisfaction [6].

It is important to note that nurses’ work methods are often shaped by various factors, such as hospital policies, leadership style, patient load, and workload [10]. Research has shown that increased nurse workloads and a lower nurse-to-patient ratio may pose risks to patient safety and negatively affect outcomes. Furthermore, when nurses face high workloads, the likelihood of errors and adverse events in patient care can increase [11]. Favorable practice environments also play a significant role in work methods, where nurses report high levels of job satisfaction and provide more attentive and dedicated care, positively impacting patient outcomes [12]. Well-organized workflows and balanced workloads reduce nurse stress and protect against burnout [13].

Evaluating work methods has a direct impact on nurses’ job satisfaction and motivation [6]. The results of this evaluation reveal nurses’ strengths and weaknesses, offering concrete information on how to direct their personal and professional development [3].

Contributing to the definition and adoption of nursing work methods should be a strategic priority to enable nurses to work more effectively, safely, and with high quality. Additionally, encouraging active participation in work processes and greater involvement in decision-making are important factors for increasing job satisfaction and improving patient care quality [14].

In alignment with the social mandate of the nursing profession, nurses are required to design and implement evidence-based care and adopt appropriate work methods that promote care quality and patient safety [15].

In this context, the Nurses’ Work Methods Assessment Scale (NWMAS) [16,17] serves as a tool to assess nurses’ work methods. The scale was developed using the Quality Standards for Nursing Care from the Portuguese Nurses Association [18] and Imogene King’s Theory of Goal Attainment as its theoretical framework [19]. This theory highlights the importance of reciprocal interactions between nurses and patients, focused on establishing shared goals through effective communication and mutual involvement in the care process. By incorporating this theoretical foundation, the NWMAS promotes a nursing practice that is person-centered and outcome-oriented, reinforcing clinical decision-making and care management. The scale has been recognized as a valid instrument for assessing the methods used by nurses in hospital settings, allowing for a standardized evaluation of nursing practices and supporting decision-making processes in nursing management [17].

Despite the existence of several tools for evaluating nursing work methods in international literature, there is currently no validated and culturally appropriate instrument available in Turkish that comprehensively captures the theoretical, practical, and organizational aspects of nurses’ work methods in hospital settings. Most existing tools either emphasize task distribution and workflow efficiency or fail to reflect local organizational structures and cultural dynamics. In this context, the Nurses’ Work Methods Assessment Scale (NWMAS) stands out as a theoretically grounded and practice-oriented instrument that offers a holistic view of nursing work organization. Given the importance of improving care quality, nurse satisfaction, and patient safety, there is a clear need for a valid and reliable tool that can assess how nurses organize their work in Turkish healthcare institutions. This study aims to address this gap by translating, adapting, and validating the NWMAS for the Turkish context.

In the current Turkish nursing literature, there is a notable gap in standardized tools that comprehensively assess nurses’ work methods from both theoretical and organizational perspectives. Existing studies tend to focus on isolated aspects such as workload, time management, or task distribution, often neglecting the broader conceptual and relational dimensions of nursing practice. Moreover, the few instruments used in previous research have either not undergone rigorous psychometric validation or lack cultural relevance to the Turkish healthcare context. This absence limits the ability of nursing administrators, educators, and researchers in Turkey to systematically evaluate and improve work method organization within clinical practice. By adapting and validating the NWMAS—a scale grounded in both theoretical nursing models and clinical standards—this study addresses this gap and provides a culturally appropriate tool that supports evidence-based nursing management and enhances care quality.

## 2. Materials and Methods

### 2.1. Design, Samples, and Settings

A methodological study was conducted with a non-probabilistic convenience sample, following the recommendations of Streiner and Norman (2018) [20]. The theoretical framework adopted was the taxonomy, terminology, and definition of health measures from the International Consensus-based Standards for the Selection of Health Measurement Instruments (COSMIN) [21]. The inclusion criteria included being nurses with at least one year of professional experience who volunteered to participate. Nurses who were not in caregiving roles (due to absence or other assignments) were excluded.

The sample size was determined based on the total population of 256 nurses working at the hospital during the study period. A minimum sample size of 154 was calculated using the following formula for finite populations:

n = p (100 − p)z^2^/E^2^

n: Sample size, p: The percentage of occurrence of a condition or situation, z: Confidence level, E: Margin of error (accuracy level) or the risk level ignored by the researcher.

Where n is the required sample size, n = 256 (population), z = 1.96 (for 95% confidence), p = 0.5 (maximum variability assumed), and d = 0.05 (margin of error). Based on this calculation, a minimum of 154 participants was required. A total of 209 nurses participated in the study, exceeding the minimum threshold and thereby increasing the power and stability of psychometric analyses.

### 2.2. Instrument

The Nurses’ Work Methods Assessment Scale (NWMAS) is composed of 25 items categorized into five factors. Responses are recorded using a five-point Likert scale, where 1 signifies “never”, 2 indicates “rarely”, 3 represents “sometimes”, 4 denotes “often”, and 5 stands for “always.” The dimension with the highest average score represents the most commonly utilized work method among the nurses. For example, a high average score in the “individual management” dimension suggests that this method is the most frequently applied by the participants. Additionally, a separate questionnaire was employed to gather information on sociodemographic and professional characteristics, including gender, age, educational background, marital status, years of experience, field of specialization, intentional career choice, and job satisfaction.

### 2.3. Data Collection

The data of the study were collected from nurses working in a public hospital in the Central Anatolia Region of Turkey between 1 June and 19 July 2024. The researcher personally distributed informed consent forms and questionnaires to nurses who chose to participate voluntarily. Participants were briefed on the study’s goals and procedures and provided their consent by signing the forms. The consent forms and questionnaires were collected separately to ensure confidentiality. All information obtained was used solely for this study and handled with strict confidentiality.

### 2.4. Data Analysis

The data of the study were collected from nurses working in a public hospital in the Central Anatolia Region of Turkey between 1 June and 19 July 2024. To adapt the “Nurses’ Work Method Assessment Scale (NWMAS)” [16,17] into Turkish, various methods were employed, including expert consultations, Bartlett’s test of sphericity, Kaiser–Meyer–Olkin (KMO) test, exploratory factor analysis, principal component analysis, and confirmatory factor analysis, to assess content and construct validity. For evaluating the scale’s reliability, internal consistency and homogeneity were assessed through Cronbach’s α coefficient, Pearson correlation analysis, item-total score correlation, and test-retest analysis.

The adaptation process of the scale consists of several steps. Initially, permission was requested via email from the author to adapt and use the NWMAS in Turkish. To ensure linguistic accuracy, the translation-back translation method was employed. Three bilingual experts translated the scale from Portuguese to Turkish, followed by another three bilingual experts who back-translated it into Portuguese. A single consensus form was created by reconciling the translations among the language experts. In the next stage, the linguistic and conceptual appropriateness of the scale was reviewed by experts [22]. To verify the linguistic and conceptual adequacy of the Turkish version, evaluations were conducted by a total of eight experts: One health management specialist, one nursing management specialist, four nurses, and two medical history and ethics specialists. The relevance, clarity, specificity, and face validity of the measurement items were assessed using the Davis method. The Expert Evaluation Form, prepared by the researchers to collect expert opinions, was utilized. According to this method, each item was rated on a scale from 1 to 4 (1 = not relevant, 2 = somewhat relevant, 3 = relevant, and 4 = highly relevant). The Content Validity Index (CVI) was calculated by dividing the number of experts who rated an item as 3 or 4 by the total number of experts. The criterion for an item’s adequacy in terms of content validity was set at 0.80. As a result of this evaluation, the item under the Functional Management factor, “FWM4 Sinto que o meu trabalho é reconhecido, através da realização de intervenções padronizadas—I feel that my work is appreciated by performing standardized interventions”, was removed from the scale because its CVI was below 0.80. Except for this item, all other items had a Content Validity Index (CVI) above 0.80 [23].

Following this, a pilot study was conducted with 61 nurses who were not part of the main study sample. Using principal component analysis with varimax rotation, a revised measurement tool consisting of 24 items across 5 factors was developed. The factor loading ranged from 0.51 to 0.77, the KMO value was 0.71, and Cronbach’s alpha was 0.87. Post-pilot, construct validity and reliability analyses of the scale were completed.

Test-retest reliability assesses the consistency of test results across repeated measurements. To evaluate this, correlation analysis is employed to compare the results from the initial and subsequent administrations. A correlation coefficient nearing 1 signifies high test-retest reliability. In this study, the correlations between the first and second administrations for each factor were statistically significant (*p* = 0.000). The scale was re-administered to 30 participants after a two-week interval, yielding test-retest correlations of 0.981 for the overall scale, 0.975 for the Team Management (F1) factor, 0.980 for the Nurse Guidance Management (F2) factor, 0.981 for the Individual Management (F3) factor, 0.960 for the Functional Management (F4) factor, and 0.952 for the Good Work Environment (F5) factor. These results indicate that the scale and its individual factors exhibit high external reliability and maintain a stable structure.

### 2.5. Ethical Considerations

Approval was obtained from the relevant university’s ethics committee on [15 May 2024] with [E-25403353-050.04-240113630]. Additionally, written permission was received from the original authors who developed the scale.

## 3. Results

Of the 209 nurses who participated in the study (Table 1), 87.1% were female, 53.6% were married, 74.2% held a postgraduate degree, 59.8% were working in Internal Medicine, 82.8% had consciously chosen their profession, and 80.9% reported practicing their profession with enthusiasm. The average age of the participants was 31, and the average number of years in the profession was 8.

The responses from 209 participating nurses were analyzed (Table 2). The KMO (Kaiser–Meyer–Olkin) measure was found to be 0.83. To examine factor analysis, two of the most commonly used statistical techniques were employed: Principal Component Analysis (PCA) and Varimax rotation. The test-retest analysis revealed that the correlation between the first and last tests was 0.97 for F1, 0.98 for F2, 0.98 for F3, 0.96 for F4, 0.95 for F5, and 0.98 for the overall NWMAS, indicating a strong relationship over time. The overall Cronbach’s alpha value for the scale was found to be 0.87, while Cronbach’s alpha values for the individual factors were as follows: F1 = 0.80, F2 = 0.82, F3 = 0.79, F4 = 0.66, and F5 = 0.52. The overall scale score for the nurses’ responses was 3.88 ± 0.458, with the highest averages found in “individual management” at 4.33 ± 0.533 and “good work environment” at 4.33 ± 0.696 (Table 3).

The exploratory factor analysis revealed that the scale consists of a five-factor structure and the construct validity was tested through confirmatory factor analysis. The five-factor structure was confirmed by confirmatory factor analysis, demonstrating that the items within each factor adequately supported the respective factors. The Composite Reliability (CR) values were found to be above 0.60, and the Average Variance Extracted (AVE) values exceeded 0.30. The correlations between the factors ranged from 0.329 to 1.000 (Table 4).

The fit indices of the confirmatory factor analysis are at an acceptable and excellent fit level (Table 5).

**Figure 1:**
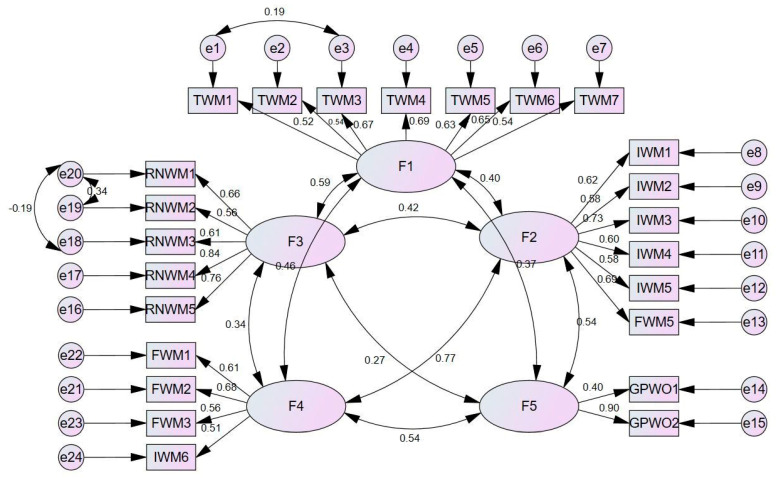
Path diagram of the confirmatory factor analysis.

Below is the path diagram that synthesizes the confirmatory analysis of the scale (Figure 1).

The findings of this study support the successful linguistic and cultural adaptation of the NWMAS to the Turkish nursing context. The five-factor structure identified through exploratory and confirmatory factor analyses aligns closely with the original scale, suggesting that the fundamental constructs of nurses’ work methods are conceptually consistent across cultural contexts. This supports the initial objective of adapting the scale for use in Turkish healthcare settings.

The high internal consistency values (Cronbach’s alpha = 0.87) indicate that the Turkish version of the scale reliably measures nurses’ work methods. Particularly, the “Individual Management” and “Team Work” dimensions showed the highest mean scores, suggesting that Turkish nurses frequently adopt these approaches in organizing their care practices. This aligns with healthcare models in Turkey, where nurses often rely on both personal initiative and team collaboration in complex hospital environments.

The findings also reveal a strong test-retest reliability (r = 0.981 overall), which underscores the temporal stability of the adapted instrument and reinforces its potential for repeated use in longitudinal or pre-post evaluation studies in nursing management. The removal of one item due to cultural incompatibility further emphasizes the importance of culturally sensitive scale adaptation and demonstrates that the adaptation process effectively filtered content not applicable to Turkish nursing practice.

Importantly, the study contributes to the broader goal of improving nursing practice by providing a standardized tool that enables hospital administrators and researchers to evaluate how nurses organize their work. This is particularly significant in light of global efforts to link nursing work structures to outcomes such as job satisfaction, patient safety, and quality of care—key issues that the scale can now help explore within Turkish healthcare institutions.

## 4. Discussion

The NWMAS, developed to evaluate the work methods of nurses, is an important measurement tool with internationally accepted validity. In this study, the adaptation of the scale into Turkish and its validity and reliability analyses were conducted, and the results were evaluated by comparing them with the literature.

The process of adapting the scale into Turkish considered Language and Cultural Adaptation as a critical phase. Language experts and nursing professionals in the field carried out the translation process, ensuring consistency among the translations. Similar studies in the literature indicate that considering cultural awareness during the adaptation of a measurement tool to another language positively impacts its validity and reliability [22,24]. Accordingly, attention was given to achieving cultural and linguistic adaptation during the translation of NWMAS into Turkish.

In the validity and reliability analysis conducted for the Turkish version, the internal consistency analysis revealed that the total Cronbach’s Alpha coefficient of the scale was 0.87, indicating high internal consistency and suggesting that it can be used as a reliable tool. Literature indicates that Cronbach’s Alpha coefficients of 0.70 and above demonstrate that a scale has a reliable structure [25]. The results obtained in this study are consistent with those found in similar validity and reliability studies [6].

Content validity was evaluated through consultations with eight experts. Their feedback was analyzed using the Content Validity Index (CVI). A CVI value greater than 0.80 indicates consensus among the experts [24,26]. In this study, CVI values ranged between 0.87 and 1.00, demonstrating a strong agreement among the experts. This consensus confirms that the scale effectively measures the intended construct and maintains content validity.

Content validity was evaluated through consultations with 7 experts. Their feedback was analyzed using the Content Validity Index (CVI). A CVI value greater than 0.80 signifies consensus among the experts [24,26]. In this study, the CVI values ranged from 0.85 to 0.95, indicating strong agreement among the experts. This consensus confirms that the scale effectively measures the intended construct and upholds content validity.

The Kaiser–Meyer–Olkin (KMO) test and Bartlett’s Sphericity Test were utilized to assess construct validity. KMO values range from 0.00 to 1.00, with values ≥ 0.70 generally considered acceptable [27]. In this study, the KMO value was 0.83, which is satisfactory. Bartlett’s Sphericity Test yielded a value of 1793.650, indicating statistical significance (*p* = 0.000). This result confirms that the dataset and sample size are appropriate for factor analysis (Table 1).

To determine the number of factors, eigenvalues greater than or equal to 1 were examined, revealing that the scale is composed of five factors: Team management, nurse guidance management, individual management, functional management, and good work environment. This aligns with the five-factor structure of the original scale [17]. However, there were discrepancies in item categorization. The item originally under Functional Management, “O resultado dos cuidados de enfermagem que presto está direcionado para o cumprimento das intervenções de enfermagem” (“The outcome of the nursing care I provide is directed towards the implementation of nursing interventions”), was reclassified under Individual Management. Conversely, the item from Individual Management, “Durante o planeamento e a implementação das intervenções de enfermagem, garanto sempre o envolvimento do cuidador/familiar cuidador” (“During the planning and implementation of nursing interventions, I always ensure the involvement of the caregiver/family caregiver”), was reclassified under Functional Management. To address these issues, a one-hour focus group discussion was conducted with 7 experts. Given the conceptual overlap between Individual Management and Functional Management and the lack of conceptual problems, it was deemed appropriate to place the items in the factor where their loadings were most suitable.

In the exploratory factor analysis, the 5-factor model accounted for 55.652% of the total variance. For multi-factor scales, it is generally expected that the explained variance exceeds 40%, with a higher variance indicating stronger construct validity. A variance explained between 40% and 60% is deemed adequate for such scales [28,29]. The amount of explained variance suggests robust construct validity. The original scale also reported a similar total variance of 55.3% [17]. Factor inclusion was based on factor loadings, which should be at least 0.30 [30]. In this study, factor loadings ranged from 0.47 to 0.78, while the original scale showed factor loadings between 0.44 and 0.88 [17]. The fact that all items had factor loadings greater than 0.30 indicates a robust factor structure for the scale.

Confirmatory factor analysis (CFA) was conducted to validate the structure identified in the exploratory factor analysis [31]. The CFA confirmed that the scale, like the original, consists of 5 factors. For the 5-factor CFA, all factor loadings were greater than 0.30. The fit indices were χ2/df = 1.89, Root Mean Square Error of Approximation (RMSEA) = 0.064, RMR = 0.06, GFI = 0.86, PGFI = 0.68, and PNFI = 0.66. According to the literature, models with χ2/df < 5 and RMSEA < 0.08 are considered to have a good fit [28,30] (Table 2). The other fit indices for the scale were CFI = 0.87, IFI = 0.87, TLI = 0.85, and MECVI = 2.83. These results align closely with those of the original scale, where CFI = 0.88, GFI = 0.87, AGFI = 0.84, IFI = 0.88, TLI = 0.859, RMR = 0.077, RMSEA = 0.065, and MECVI = 2.33 [17]. The CFA results confirm that the model fits the data well, the five-factor structure is validated, and the items are appropriately associated with their respective factors. Both the exploratory and confirmatory factor analyses support the construct validity of the scale, affirming its status as a valid measurement tool.

The convergent validity of the scale was examined as follows: For the Team Management (F1) factor, CR was 0.80 and AVE was 0.38; for the Nurse Guidance Management (F2) factor, CR was 0.81 and AVE was 0.48; for the Individual Management (F3) factor, CR was 0.80 and AVE was 0.40; for the Functional Management (F4) factor, CR was 0.69 and AVE was 0.35; and for the Good Work Environment (F5) factor, CR was 0.62 and AVE was 0.49. Although the literature suggests that AVE values should be higher than 0.50 and CR values should be higher than 0.70, Maroco (2021) indicates that convergent validity is achieved if CR is higher than 0.60, even if AVE is lower than 0.50. The scale appears to have established convergent validity [32].

In the literature, Cronbach’s α is reported to range between 0.00 and 1.00, with reliability increasing as the value approaches 1.00. Values of 0.50 and above are considered acceptable, while values above 0.70 are desired [26,27]. In this study, the overall Cronbach’s α of the scale was 0.87, with factor-specific Cronbach’s α values being F1 0.80, F2 0.82, F3 0.79, F4 0.66, and F5 0.52. The low number of items in the subdimensions may be the reason for this decrease. In the literature, it is stated that as the number of items in a scale decreases, the Cronbach’s Alpha value may also be lower [33]. Although two factors had values below 0.70, these are still considered acceptable, and the high overall Cronbach’s α indicates strong internal consistency (Table 1). In the original scale, the last two factors also had lower Cronbach’s α values, but the overall Cronbach’s α was found to be 0.84 [17]. These results suggest that the Turkish version of the NWMAS scale is a reliable measurement tool for evaluating nurses’ work methods.

Item-total correlation analysis shows the correlation between scores obtained from the scale items and the total scale score. This value should be >0.30 [25,34,35]. In this study, the correlation for all items, except one, is greater than 0.30. The item with the lowest correlation did not significantly affect the Cronbach alpha value and thus was not removed from the analysis (Table 2).

The adaptation and validation of the NWMAS for the Turkish context offer significant benefits, such as the cultural adaptation of the instrument, reflecting the working methods of Turkish nurses in hospital settings. Furthermore, it allows for a better understanding of nurses’ work organization, enabling the collection of relevant data that can impact patient safety and care quality, as well as professional satisfaction. It also facilitates the identification of training needs and the development of more effective training programs tailored to nurses’ specific requirements.

### Limitations

This study presents several limitations that must be considered when interpreting the findings. First, a non-probabilistic convenience sampling method was employed, which may limit the generalizability of the results. The participants, although diverse in terms of clinical departments and backgrounds, may not represent the broader nursing population in Turkey. Future studies using probabilistic or stratified sampling techniques could enhance representativeness and external validity.

Second, the use of the same sample for both the exploratory and confirmatory factor analyses introduces the risk of overfitting. Although this approach allowed us to validate the factor structure within the same dataset, it may have led to an inflated model fit. Ideally, the confirmatory factor analysis (CFA) should be conducted on an independent sample to ensure the stability of the scale’s structure. We addressed this concern by interpreting the CFA results diagnostically and plan to test the model in future studies using different samples.

Third, the cultural context of the Turkish healthcare system may have influenced how certain items were interpreted. One item was removed due to cultural incompatibility, which suggests that the scale may require further adaptation for subgroups within Turkey or other regions with different healthcare delivery models.

Additionally, self-reporting bias may have affected the accuracy of responses. Participants could have responded in a socially desirable manner, particularly in relation to questions about professional enthusiasm, autonomy, or organizational practices. While anonymity was maintained, future studies might incorporate triangulated methods (e.g., observational data, supervisor assessments) to mitigate such bias.

Finally, test-retest reliability was assessed on a small subsample of 30 participants, which limits the robustness of the temporal stability findings. Larger samples would provide stronger evidence of the scale’s reliability over time.

Despite these limitations, the study provides important insights into the adaptation and validation of the NWMAS for Turkish nurses and sets the groundwork for future research in diverse clinical contexts.

## 5. Conclusions

According to the analysis results, the Turkish version of the scale consists of five factors, similar to the original version. However, due to one item not fitting the Turkish cultural context, as advised by expert opinions, the scale was reduced from 25 to 24 items. Structural validity analyses confirmed that the scale items are grouped under five factors. The Turkish Cronbach α internal consistency coefficient, item-total correlation, and test-retest analysis are at satisfactory levels. The “Nurses’ Work Method Assessment Scale (NWMAS)” has been validated as suitable for the Turkish culture. It can be used to assess nurses’ work methods within the Turkish cultural context. From an organizational and public policy perspective, the use of the NWMAS adapted to Turkey can optimize resource management and improve operational efficiency in healthcare services. Additionally, it provides a valuable database for shaping policies and regulations related to nurses’ working conditions. This contributes not only to the retention and well-being of nursing professionals but also to a more sustainable and efficient healthcare system in the Turkish context.

The results of this study provide a significant contribution to understanding nurses’ work methods. However, further testing of the scale’s validity on a broader sample and in different healthcare settings is needed. Additionally, examining how data obtained from this scale relate to nurses’ job satisfaction, burnout, and quality of care could be an important area for future research.

These results not only confirm the scale’s psychometric adequacy but also affirm its alignment with the study’s original objective: To provide a culturally relevant and theoretically grounded instrument for evaluating Turkish nurses’ work methods. The validated scale can now serve as a foundation for future empirical studies exploring how work methods influence nurse performance, patient outcomes, and organizational efficiency.

## Figures and Tables

**Table 1:** Participants’ sociodemographic and professional descriptive measures.

Socio-Demographic Characteristics		n (%)
Gender	Female	182 (87.1)
	Male	27 (12.9)
Marital Status	Married	112 (53.6)
	Single	97 (46.4)
Educational Status	Associate Degree	12 (5.7)
	Bachelor’s Degree	42 (20.1)
	Postgraduate Degree	155 (74.2)
Department of Employment	Internal Medical Sciences	125 (59.8)
	Surgical Medical Sciences	84 (40.2)
Did you consciously choose your profession?	Yes	173 (82.8)
	No	36 (17.2)
Are you doing your job willingly?	Yes	169 (80.9)
	No	40 (19.1)
Years of Working in Nursing	$\bar{x}$ ± s.d.: 8 ± 8779	
Age	$\bar{x}$ ± s.d.: 31 ± 8265	

$\bar{x}$/s.d.—Mean/Standard Deviation.

**Table 2:** Exploratory Factor Analysis, Factor Loading, Item Total Correlation, KMO Bartlett’s, Test-Retest, Cronbach’s Alpha, Item Means, Factor Means.

Factor		Item	Factor Load	Corrected Item-Total Correlation	Item α	Variance Explained %	Cumulative Variance %	Test and Retest *r*	α	Factor $\bar{x}$/s.d.
Team work method—F1	The nursing care I provide is guided and supervised by a nurse designated as the team leader/head nurse.	TWM1	0.699	0.386	0.872	13.385	55.652	0.975	0.80	4.03 ± 0.48
	Daily meetings are held between nurses and nurse leaders/team heads to ensure continuity of care and to discuss the nursing care to be provided to patients.	TWM2	0.637	0.379	0.872					
	There is a team leader who is responsible for ensuring the delivery of high-quality and safe nursing care, utilizing leadership, control, and strategic planning.	TWM3	0.781	0.479	0.869					
	Continuity of care is maintained in daily practice through discussions of nursing care plans between lead nurses and other nurses.	TWM4	0.751	0.469	0.869					
	Patients’ care needs are met by a team of nurses with varying levels of competence.	TWM5	0.537	0.522	0.867					
	In each shift, the planning and implementation of care are carried out by a group of nurses.	TWM6	0.532	0.605	0.865					
	The team leader discusses and determines strategies with the nurses for involving family members in the planning and implementation of care.	TWM7	0.512	0.439	0.870					
Reference nurse work method—F2	Each patient is assigned a primary (reference) nurse throughout the hospitalization process, from admission to discharge. When this nurse is not on duty, other nurses take over their responsibilities.	RNWM1	0.687	0.513	0.868	12.884		0.980	0.82	4.03 ± 0.48
	In the absence of the primary (reference) nurse, the patient’s care is provided by other nurses.	RNWM2	0.672	0.434	0.870					
	Whenever possible, the patients I admit to the unit are assigned under my responsibility.	RNWM3	0.736	0.402	0.871					
	The reference nurse plans and evaluates the care provided by other nurses and proposes changes in the planning and implementation of care from the patient’s admission to discharge.	RNWM4	0.760	0.610	0.864					
	Any revisions to the care plan must be approved by the patient’s assigned (reference) nurse.	RWM5	0.739	0.560	0.866					
Individual work method—F3	While organizing nursing care, I prioritize the patient and ensure their active participation in the care process.	IWM1	0.694	0.451	0.870	12.193		0.981	0.79	4.33 ± 0.53
	I believe that caring for the same patients throughout a shift leads to more humane and personalized nursing services.	IWM2	0.476	0.394	0.871					
	The nursing care I provide is directed toward comprehensive patient care.	IWM3	0.724	0.445	0.870					
	In each shift, I evaluate the outcomes of nursing interventions in order to revise the care plan as needed.	IWM4	0.718	0.462	0.869					
	I always involve the patient in the planning and implementation of nursing interventions.	IWM5	0.549	0.414	0.871					
	The outcome of the nursing care I provide is directed toward fulfillment of nursing interventions.	FWM5	0.647	0.363	0.872					
Functional work method—F4	During my shift, I perform nursing interventions for all patients admitted to the unit.	FWM1	0.647	0.363	0.872	10.762		0.960	0.66	4.19 ± 0.56
	When a patient is admitted to the unit, I share the nursing interventions with other nurses to facilitate the work.	FWM2	0.667	0.446	0.870					
	My work focuses on predefined and standardized procedures.	FWM3	0.495	0.395	0.871					
	I always ensure the involvement of caregivers and/or family members in the planning and implementation of nursing interventions.	IWM6	0.715	0.575	0.871					
Good practices in work organization—F5	During a shift, I take full responsibility for planning and delivering care for the patients assigned exclusively to me.	GPWO1	0.772	0.223	0.876	6.428		0.952	0.52	4.33 ± 0.69
	The focus of my work is the planning and implementation of patient care during the shift.	GPWO2	0.682	0.436	0.870					
NWMAS		KMO Barlet’s: 0.83; χ2: 1793.65; *p*: 0.000						0.981	0.87	3.88 ± 0.45

$\bar{x}$/s.d.—Mean/Standard Deviation.

**Table 3:** An exploratory factor analysis.

	Component				
	1	2	3	4	5
TWM1	**0.699**	−0.013	0.156	0.045	0.066
TWM2	**0.637**	0.187	0.032	0.005	0.082
TWM3	**0.781**	0.042	0.114	0.036	0.136
TWM4	**0.751**	0.115	−0.025	0.182	0.017
TWM5	**0.537**	0.348	0.043	0.169	0.257
TWM6	**0.532**	0.277	0.315	0.083	0.274
TWM7	**0.512**	0.253	0.004	0.301	0.117
RNWM1	0.150	**0.687**	0.290	0.007	0.015
RNWM2	0.109	**0.672**	0.143	0.055	0.202
RNWM3	0.081	**0.736**	0.174	0.276	0.036
RNWM4	0.327	**0.760**	0.117	0.086	0.019
RNWM5	0.112	**0.739**	0.304	0.033	0.129
IWM1	0.101	0.138	**0.694**	0.111	0.111
IWM2	0.065	0.177	**0.476**	0.414	0.042
IWM3	0.064	0.024	**0.724**	0.269	0.145
IWM4	0.075	0.225	**0.718**	0.047	0.088
IWM5	0.090	0.055	**0.549**	0.427	0.125
IWM6	0.093	0.176	**0.096**	0.715	0.126
FWM1	0.167	0.113	0.187	**0.647**	0.151
FWM2	0.094	0.067	0.185	**0.667**	0.257
FWM3	0.126	0.062	0.246	**0.495**	0.189
FWM5	0.140	0.063	0.526	**0.449**	0.194
GPWO1	0.096	0.174	0.061	0.077	**0.772**
GPWO2	0.164	0.040	0.277	0.258	**0.682**

**Table 4:** Confirmatory Factor Analysis. CR. AVE Inter-factor correlation.

Factor	Item	Factor Load	Standard Error	t	*p*	CR	AVE	Correlation				
								F1	F2	F3	F4	F5
Team work method—F1	TWM1	0.521	-	-	<0.05	0.80	0.38					
	TWM 2	0.540	0.19	5.75	<0.05							
	TWM 3	0.675	0.19	7.14	<0.05							
	TWM 4	0.694	0.23	6.59	<0.05							
	TWM 5	0.628	0.20	5.99	<0.05							
	TWM 6	0.650	0.20	6.11	<0.05							
	TWM 7	0.544	0.20	5.59	<0.05							
Reference nurse work method—F2	RNWM1	0.660	-	-	<0.05	0.81	0.48	1.00				
	RNWM 2	0.558	0.08	8.52	<0.05							
	RNWM 3	0.612	0.10	6.9	<0.05							
	RNWM 4	0.841	0.12	9.22	<0.05							
	RNWM 5	0.758	0.12	8.87	<0.05							
Individual work method—F3	IWM1	0.615	-	-	<0.05	0.80	0.40	0.70	0.70			
	IWM 2	0.577	0.16	6.82	<0.05							
	IWM 3	0.730	0.17	8.01	<0.05							
	IWM 4	0.596	0.17	7.05	<0.05							
	IWM 5	0.582	0.16	6.83	<0.05							
	**FWM 5**	**0.688**	**0.14**	**7.5**	**<0.05**							
Functional work method—F4	FWM1	0.607	-	-	<0.05	0.69	0.35	0.64	0.64	0.57		
	FWM2	0.680	0.19	7.12	<0.05							
	FWM3	0.565	0.19	5.97	<0.05							
	**IWM6**	**0.514**	**0.22**	**5.61**	**<0.05**							
Good practices in work organization—F5	GPWO1	0.403	-	-	<0.05	0.62	0.49	0.46	0.46	0.37	0.32	-
	GPWO2	0.900	0.55	3.26	<0.05							

CR—Composite Reliability; AVE—Average Variance Extracted.

**Table 5:** Fit Index values.

Fit Index	NWMAS	Suitable	Acceptable	Result
χ2/df	1.89	<2	<5	Perfect fit
RMSEA	0.06	<0.05	<0.08	Acceptable fit
RMR	0.06	<0.05	<0.08	Acceptable fit
GFI	0.86	>0.95	>0.85	Acceptable fit
PGFI	0.68	>0.89	>0.50	Acceptable fit
PNFI	0.66	>0.89	>0.50	Acceptable fit

df—Degrees of Freedom; RMSEA (Root Mean Square Error of Approximation); RMR (Root Mean Square Residual); GFI (Goodness-of-Fit Index); PGFI (Parsimony Goodness-of-Fit Index); PNFI (Parsimony Normed Fit Index).

## Data Availability

The data presented in this study is available on request from the corresponding author. The data are not publicly available due to ethical restrictions.

## Public Involvement Statement

No public involvement in any aspect of this research.

## Guidelines and Standards Statement

This manuscript was prepared on the basis of COSMIN for methodological study research.
